# Five Decades of Innovation—Tailored Breast Cancer Treatment: 1976–2026

**DOI:** 10.1002/wjs.70381

**Published:** 2026-04-25

**Authors:** Ipshita Prakash, Pooja Ramakant, Sandra Krishnan, Sharon W. W. Chan, Tadahiko Shien, Kavitha Daester, Michael Douek, Mee Hoong See, Jacqueline Jeruss, Cheng‐Har Yip, Ines Buccimazza

**Affiliations:** ^1^ Departments of Surgery & Oncology McGill University Montreal Quebec Canada; ^2^ Lady Davis Institute Segal Cancer Center Jewish General Hospital Montreal Quebec Canada; ^3^ Endocrine and Breast Surgery Department King Georges Medical University Lucknow India; ^4^ Sydney Adventist Hospital Westmead Private Hospital Sydney New South Wales Australia; ^5^ Department of Surgery United Christian Hospital KEC Breast Centre HA The University of Hong Kong The Chinese University of Hong Kong Hong Kong China; ^6^ Department of Breast and Endocrine Surgery Okayama University Hospital Okayama Japan; ^7^ Affidea BrustCare Brust‐Zentrum Zürich Zürich Switzerland; ^8^ Nuffield Department of Surgical Sciences University of Oxford Oxford UK; ^9^ Breast and Endocrine Surgery Unit General Surgery Division Department of Surgery Faculty of Medicine Kuala Lumpur Malaysia; ^10^ Departments of Surgery, Pathology, and Biomedical Engineering University of Michigan Ann Arbor Michigan USA; ^11^ PJ Integrated Centre for Advanced Surgery and Oncology (PICASO Hospital) University Malaya Kuala Lumpur Malaysia; ^12^ Department of Surgery Nelson R Mandela School of Medicine University of KwaZulu‐Natal Durban South Africa

## 1 Introduction

Breast cancer treatment has undergone a major paradigm shift over the past 5 decades. Treatment has become more personalized, tailored, and less invasive. Deviations from previous gold standards (radical mastectomy and routine axillary dissection) due to robust evidence that “less is more” and a more sophisticated understanding of breast cancer biology has resulted in a de‐escalation of many breast cancer treatment modalities, a movement led by breast surgeons.

In this manuscript, experts from Breast Surgery International will highlight five breast cancer surgery innovations which have transformed the field into a precise science, with improved outcomes.

## 2 The Axilla in Breast Cancer: Five Decades of Surgical De‐Escalation

In the past 5 decades, the axillary management of breast cancer has undergone significant de‐escalation driven by improvements in the understanding of disease biology and technological innovations in diagnostic and therapeutic approaches. Advances in breast imaging, systemic therapies, radiation therapy, and surgical techniques have shifted the role of axillary surgery from a therapeutic intervention to a staging procedure. Axillary lymph node dissection (ALND)—the Halstedian legacy and standard of care for breast cancer in the 1980s—gave way to sentinel lymph node biopsy (SLNB), which was widely adopted in the 1990s [1, 2]. Since that time, further de‐escalation strategies, including limited axillary surgery after neoadjuvant systemic therapy, targeted axillary surgery, and omission of axillary surgery altogether, have emerged as viable options for selected patients [1, 2]. In this era of precision oncology and personalized medicine, disease biology and shared‐decision making have taken center‐stage. Contemporary axillary management aims to balance oncologic safety with surgical morbidity and patient‐centered outcomes.

The advent and evolution of SLNB for patients with early‐stage breast cancer and clinically negative axillae in the 1980s–90s was initially met with considerable resistance [3, 4]. Several randomized trials comparing SLNB to ALND were completed before SLNB was accepted as the new standard of care for this population. The landmark National Surgical Adjuvant Breast and Bowel Program (NSABP)‐B32 and Milanese trials [3, 4] confirmed the feasibility of SLNB. Subsequently, the American College of Surgeons Oncology Group Z0011, AMAROS, and OTOASOR trials confirmed the safety of this surgical approach by showing equivalent axillary recurrence rates and survival outcomes for patients undergoing SLNB versus ALND in the setting of clinically node negative sentinel node positive breast cancer [5, 6, 7]. Recently, new trials in the context of modern breast cancer management, such as SENOMAC and SINODAR‐ONE, have reaffirmed these results [8, 9].

The successful implementation of SLNB in the primary surgery setting in the 1990s–2000s prompted the evaluation and adoption SLNB in the postneoadjuvant context in the 2010s. Trials, including Alliance Z1071, SENTINA, SN‐FNAC, and GANEA‐2, demonstrated acceptable false‐negative rates especially when technical refinements were applied [10, 11, 12, 13]. The incorporation of improved localization techniques led to the incorporation of SLNB and targeted axillary surgery (i.e., removal of the localized positive lymph node along with the sentinel nodes) as the new standard approach for patients with clinically negative axillae postneoadjuvant systemic therapy [10, 11, 12, 13].

The past 5 years have marked a further evolution in axillary management, namely, omission of SLNB in the primary surgery setting for patients with clinical T1‐2 breast cancer and negative axillae by preoperative axillary ultrasonography (AUS). The randomized controlled SOUND, INSEMA, and BOOG 2013‐08 trials demonstrated the noninferiority of SLNB omission compared to SLNB in this low‐risk population [14, 15, 16]. Unlike the prolonged adoption curve seen in the 1990s, these data are being rapidly incorporated into current guidelines [17, 18].

Despite the pendulum swinging from routine ALND to selective omission of axillary surgery, ALND remains indicated for most patients with residual nodal disease postneoadjuvant systemic therapy and those with locally advanced breast cancer undergoing primary surgery. In these populations, upper extremity lymphedema rates remain high (21.5%–28%) [19]. Innovations in microsurgical techniques have led to prophylactic and therapeutic procedures, such as lymphatic microsurgical preventive healing approach (LYMPHA) and lympho‐venous bypass, being developed for lymphatic preservation, thus helping mitigate the effects of this morbid adverse event. A recent prospective trial on ALND with or without immediate lymphatic reconstruction did not find any significant difference in lymphedema rates between the two groups [20].

## 3 Oncoplastic Breast Surgery: Another Arrow in the Breast Surgeon's Quiver

The next frontier of breast cancer surgery is not doing more; it is doing better—more precise, more personal, more oncoplastic.

Sandra Krishnan, Breast Surgeon, Sydney

Oncoplastic breast surgery (OBS) has transformed the interface between oncologic safety and aesthetic restoration, adding “another arrow” to the contemporary breast surgeon's quiver. First articulated by Audretsch in the late 1990s, the discipline evolved rapidly through global pioneers, demonstrating that extensive resections with symmetrization is both oncologically and cosmetically superior [21, 22]. Breast surgeons today firmly position OBS as a natural evolution of breast‐conserving surgery, integrating volume displacement and replacement techniques within multidisciplinary care pathways [23, 24]. Five transformative impacts of OBS are illustrated as follows.

### 3.1 Expansion of Eligibility for Breast Conservation Enhancing Oncologic Outcomes

The most profound impact is for patients previously destined for mastectomy—OBS permits wide resections in locally advanced cancers [23, 25]. Systematic reviews show that OBS after neoadjuvant therapy can safely extend indications for breast conservation without compromising local control [25]. A major Swedish study of nearly 49,000 women found that breast‐conserving surgery plus radiotherapy significantly improved both overall and breast cancer‐specific survival compared to mastectomy [26]. OBS significantly expands this survival advantage.

### 3.2 Improved Margin Control and Reduced Re‐Operation Rates

Allowing larger segmental resections with immediate reshaping, OBS reduces the traditional challenge between clear margins and acceptable cosmesis. This is associated with lower positive margin and re‐excision rates [24]. Recent meta‐analyses comparing OBS with conventional BCS demonstrated reduced re‐excisions and conversion‐to‐mastectomy rates without detriment to local recurrence or survival, underscoring oncologic safety [27].

### 3.3 Systematic Elevation of Aesthetic and Patient‐Reported Outcomes

OBS explicitly embeds aesthetic planning into oncologic decision‐making. Prospective cohorts using BREAST‐Q and other PROMs demonstrated higher satisfaction with breast appearance, better body image and improved health‐related quality of life when compared with standard BCS [23, 28, 29]. This has reframed success in breast surgery as a composite of margin status, complication profile, and improved patient‐reported outcomes.

### 3.4 Reconfiguration of Multidisciplinary Services and Training

The rise of OBS has been the catalyst for new service models that incorporate reconstructive options at the first cancer discussion. National guidance from professional organizations now defines standards for oncoplastic decision‐making, case selection and outcome audit, making OBS standard of care in high‐income settings [30]. Structured fellowships have created a recognized subspecialty skillset, although global provision remains variable and a key equity challenge [31].

### 3.5 Richer Technical Armamentarium and Algorithmic Planning

The surgeon's technical palette has expanded to a graded spectrum of volume displacement (level I–II therapeutic mammoplasty and mastopexy patterns), volume replacement (perforator and muscle flaps, LICAP/TDAP, and mini‐latissimus dorsi), extreme oncoplasty to extend the indications of OBS for multifocal/multicentric tumors using innovative therapeutic mammoplasty techniques [32], and hybrid approaches, supported by quadrant‐specific atlases and decision frameworks [23, 27]. This has enabled reproducible planning, better integration with radiotherapy fields, and more consistent results.

### 3.6 Future Directions and Global Priorities

Over the next decade, OBS will be defined by evidence consolidation, worldwide dissemination, and technological integration. Data from prospective registries and comparative trials should inform shared decision‐making and guideline updates [24, 25, 27, 31]. Globally, the immediate priority is capacity‐building—expanding access to structured training, virtual mentorship, and low‐cost flap/reduction techniques tailored to resource‐constrained environments. International oncoplastic training networks and open‐access educational platforms can offer early scale‐up models [31].

The future is exciting and includes three‐dimensional imaging, digital planning, and AI‐supported outcome prediction [24]. OBS will evolve from “another arrow in the quiver” to an expectation that every woman, everywhere is offered oncologically sound surgery which also respects her form, identity, and wellbeing.

## 4 Paradigm Shifts in Local Treatment: From Routine Intervention to Selective Local Control

The widespread implementation of mammographic screening has dramatically altered the landscape of breast cancer diagnosis. One of its most notable consequences has been the marked increase in the detection of ductal carcinoma in situ (DCIS). However, this rise in DCIS incidence has not been accompanied by a proportional reduction in the incidence of advanced or metastatic breast cancer, while also raising concerns regarding overdiagnosis and overtreatment [33]. These observations have prompted a critical re‐evaluation of the role of routine surgical intervention, particularly for biologically indolent low‐risk DCIS.

Retrospective studies have suggested that breast surgery for selected low‐risk DCIS may not translate into a meaningful survival benefit, challenging long‐held assumptions that all DCIS inevitably progress to invasive disease [34]. Against this backdrop, several prospective clinical trials (COMET (USA); LORETTA (Japan); LORD (Europe); and LORIS (UK)) have been initiated to define the optimal management of low‐risk DCIS, including strategies for nonoperative management and active surveillance [35]. The COMET randomized trial demonstrated that active monitoring was noninferior to guideline‐concordant surgery with respect to short‐term ipsilateral invasive cancer risk at 2 years. In contrast, the single‐arm LORETTA trial of endocrine therapy alone did not meet its predefined primary endpoint for invasive cancer incidence, underscoring the need for longer follow‐up and careful patient selection. At present, robust evidence confirming the long‐term safety of omission of surgery is still lacking, and mature outcome data are awaited. Until such data become available, careful patient selection, shared decision‐making, and transparent discussion of uncertainties remain essential.

A similar paradigm shift has occurred in the context of de novo stage IV breast cancer. Historically, surgical resection of the primary tumor was often considered as part of routine management, driven by retrospective data suggesting a survival advantage. More recently, multiple prospective randomized trials have addressed this question rigorously [36, 37, 38, 39, 40]. These studies consistently demonstrate that early locoregional surgery for the primary tumor does not confer a significant overall survival benefit compared with systemic therapy alone. Nevertheless, after surgical resection, local control within the breast was significantly improved, highlighting an essential role for surgery in symptom prevention and quality‐of‐life preservation rather than in prolonging survival.

These findings underscore the need to refine indications for local therapy in metastatic disease. Rather than a blanket approach, future efforts should focus on identifying patient subgroups who may derive meaningful benefit from aggressive local treatment. In this context, increasing attention has been directed toward oligometastatic breast cancer, characterized by a limited number of metastatic lesions [41]. Ongoing studies are evaluating whether intensified local therapy to both the primary tumor and metastatic sites can favorably influence long‐term outcomes in this selected population.

In the era of treatment de‐escalation, a shift has also been observed in the management of patients with locally recurrent breast cancer [42]. Traditionally, patients experiencing ipsilateral breast tumor recurrence following primary breast‐conserving therapy with surgery and radiation were advised to undergo mastectomy, as repeat breast irradiation was not considered a viable option.

However, with accumulating evidence supporting the safety and feasibility of breast re‐irradiation, along with advances in oncoplastic surgical techniques, repeat breast‐conserving surgery, and re‐radiation have increasingly been performed in carefully selected patients [43]. Furthermore, recent studies have demonstrated no survival advantage for mastectomy over repeat breast conservation in this setting [44]. Therefore, in selected cases, repeat breast‐conserving approaches can be considered which may significantly improve patient quality of life without compromising oncologic outcomes or survival.

## 5 Less Is More: De‐Escalating Radiotherapy

There has been a paradigm shift in the treatment of breast cancer with breast conserving surgery regarded as standard of care provided patients received adjuvant postoperative radiotherapy [45, 46]. Attempts to de‐escalate radiotherapy have been made to reduce the size of the radiation field, reduce the number of treatment fractions, and evaluate different modes of delivering radiotherapy that can reduce the morbidity while maintaining the therapeutic effect. The UK START trials A [47] and B [48] found that a radiotherapy schedule of 41.6 Gy in 13 fractions and 40 Gy in 15 fractions, respectively, offered rates of local‐regional relapse and late adverse effects similar to the standard schedule of 50 Gy in 25 fractions. A further 5 fractions, as a boost to the tumor bed, are usually administered following the results of the EORTC boost trial [49]. The FAST‐Forward trial found that a 1‐week course of radiotherapy (26 Gy in 5 fractions) is as safe and effective as the previous 3‐week standard (40 Gy in 15 fractions) for patients with early‐stage breast cancer [50]. Intensity‐modulated radiotherapy (IMRT) can improve dose homogeneity, avoiding hot spots that can cause skin damage and reduce exposure to the heart and lungs [51].

Partial breast irradiation (PBI) is a radiotherapy technique used after breast‐conserving surgery for early‐stage breast cancer. Unlike whole‐breast irradiation (WBI), PBI targets only the tumor bed with a margin. This was based on the observation that over 90% of local recurrences from breast cancer occur at the index quadrant [52, 53], the incidence of contralateral cancer is equal to the incidence of new ipsilateral tumors; and that despite the high frequency of additional cancer foci away from the index tumor (over 60%), the incidence of new ipsilateral breast cancer is low [54, 55], suggesting that these small cancer foci are clinically insignificant.

Techniques of PBI are as follows:

1. External Beam Radiotherapy (EBRT)

The NSABP B‐39 trial compared WBI delivered in 25 daily fractions of 50 Gy over 5 weeks with 3D‐CRT (three‐dimensional conformal radiation therapy) 38.5 Gy in 10 fractions, over 5 treatment days and showed slightly higher local recurrence with PBI, but absolute difference was small [56] The FLORENCE trial demonstrated that PBI using IMRT (30 Gy/5 factions) was noninferior to WBI for local control in selected early breast cancer with less toxicity and better cosmesis [57].

2. Brachytherapy

The GEC–ESTRO randomized trial evaluated PBI using multicatheter interstitial brachytherapy delivered as 30 Gy (seven fractions) and 32 Gy (eight fractions) of high‐dose‐brachytherapy in 5 days or as 50 Gy of pulsed‐dose‐rate brachytherapy over 5 treatment days against standard WBI in early‐stage breast cancer patients after breast‐conserving surgery. Long‐term follow‐up showed that PBI are as safe and effective as whole‐breast radiotherapy in patients with low‐risk early breast cancer [58].

3. Intraoperative radiotherapy

Intraoperative radiotherapy (IORT) is delivered as a single fraction at the time of operation. The Intrabeam system delivers electron generated low energy X‐rays (50 kV maximum) into the tumor bed during breast conserving surgery. The TARGIT A randomized trial demonstrated no statistically significant difference in local recurrence‐free survival (*p* = 0.28), with a significant improvement in mortality from other causes (*p* = 0.005) [59]. The TARGIT B trial evaluated IORT as a boost to the tumor bed, reducing the duration of whole breast radiotherapy [60].

The ELIOT trial compared standard WBI with 21 Gy intraoperative radiotherapy with electrons in a single dose to the tumor bed during surgery and demonstrated a higher rate of local recurrence in the ELIOT group without any difference in overall survival [61]. The system has also been used for on‐table nipple‐areolar complex irradiation during subcutaneous nipple sparing mastectomy [62].

A recent meta‐analysis of the existing trials concluded that ipsilateral breast recurrence was not statistically significantly different between PBI and WBI. Acute AEs were less frequent with PBI [63].

The 2023 ASTRO guidelines recommend PBI for women 40 years or older, early stage, node‐negative invasive breast cancer or DCIS, ER positive status, Grade 1–2, small tumor size 2 cm, or less with negative surgical margins. It is conditionally recommended for women with Grade 3 disease, ER negative histology, or tumor size between 2 and 3 cm. PBI is not recommended for women less than 40 years old, positive lymph nodes, positive surgical margins, BRCA1/2 mutations, and extensive lymphovascular invasion. Recommended techniques are 3D‐CRT, IMRT, and multicathether brachytherapy. Intraoperative radiotherapy (IORT) is not recommended outside of studies due to higher recurrence risks [64].

Several trials have evaluated omission of radiotherapy in carefully selected patients. The CALGB 9343 trial demonstrated that women ≥ 70 years with T1N0, ER‐positive tumors receiving endocrine therapy had low local recurrence rates without radiotherapy, with no difference in overall survival [65]. The PRIME II trial demonstrated that in women ≥ 65 years with low‐risk, hormone receptor–positive early breast cancer receiving endocrine therapy, omission of whole‐breast radiotherapy led to higher local recurrence but no significant difference in overall survival, supporting selective omission of radiotherapy [66].

De‐escalation of radiotherapy in breast cancer represents a shift toward more personalized care, balancing excellent oncologic outcomes with reduced treatment toxicity. Carefully selected patients, particularly those with low‐risk disease, can safely receive less intensive radiation or even omit it altogether without compromising survival. Ongoing trials continue to refine these strategies, reinforcing that the goal is not less treatment, but the right treatment, maximizing quality of life with excellent outcomes.

## 6 Advancements in Breast Surgery: Minimally Invasive Approaches Using Endoscopic and Robotic Techniques

Minimally invasive surgery represents a major advancement in breast surgery, aiming to reduce surgical morbidity, enhance cosmetic outcomes, and maintain oncologic safety. The term *minimal access breast surgery* (MAS) is increasingly preferred as these techniques emphasize the use of remote concealed incisions rather than limiting the extent of tissue excision itself. This approach aligns with the growing expectations of patients with breast cancer for improved aesthetic outcomes and quality of life without compromising cancer control, an evolution well documented in contemporary breast surgical oncology literature [67].

The development of minimally invasive techniques mirrors the broader historical evolution of breast surgery from radical mastectomy to breast‐conserving surgery. Minimal access breast surgery gained prominence in the early 2000s, influenced by advances in laparoscopic and endoscopic surgery across other surgical specialties [68]. Minimal access breast surgery encompasses a spectrum of techniques, ranging from nonendoscopic approaches to endoscopic‐assisted and robotic‐assisted procedures. Initial nonendoscopic minimal access techniques gradually evolved into endoscopic‐assisted approaches, which utilize gasless or insufflation based methods, single or multiport access, and high definition imaging to enable improved visualization of surgical planes and precise dissection through remote incision placement [69]. Robotic‐assisted breast surgery, particularly robotic nipple‐sparing mastectomy (R‐NSM), represents the most advanced form of minimal access breast surgery, offering further enhanced precision, reproducibility, superior dexterity, tremor filtration, and three‐dimensional visualization compared with conventional or endoscopic approaches [67, 70].

Robotic breast surgery employs computer‐assisted platforms that translate the surgeon's hand movements into precise stable instrument actions while providing high‐definition three‐dimensional visualization and tenfold image magnification. The da Vinci Surgical System is the most widely used platform; however, other systems, such as Senhance, Versius, Mazor X, and microsurgical platforms, including MUSA and Symani, have been developed, offering features such as haptic feedback, portability, navigation capabilities, and advanced instrumentation [71]. These robotic technologies support a range of procedures including nipple‐sparing mastectomy, sentinel lymph node biopsy, and both implant‐based and autologous breast reconstruction [71, 72].

R‐NSM is associated with less nipple necrosis and Grade 3 complications compared to conventional nipple sparing mastectomy (C‐NSM), though operative time and length of stay is increased [73, 74, 75]A randomized controlled trial showed that R‐NSM is associated with higher satisfaction, physical, psychosocial, and sexual well‐being [76]. Criteria for R‐NSM with immediate breast reconstruction (IBR) have been established with an ongoing trial confirming the long‐term oncological safety and cost‐effectiveness of this surgical approach [77].

In addition, robotic surgery offers ergonomic benefits for surgeons by reducing physical strain and fatigue during prolonged procedures [78]. In the reconstructive setting, robotic techniques have been associated with reduced postoperative pain, lower rates of nipple–areolar complex necrosis, and higher aesthetic satisfaction in appropriately selected patients [77].

Despite these advantages, several challenges limit the widespread adoption of minimal access and robotic breast surgery. These procedures are often associated with longer operative times, particularly during the learning curve, and require intensive training. High acquisition and maintenance costs of robotic platforms, limited access to technology, and a shortage of trained personnel further restrict implementation, especially in resource‐limited settings. Additional concerns include reduced tactile feedback and the potential for technical malfunction. Ongoing research is required to establish long‐term oncologic safety and cost‐effectiveness [70, 72].

The future of minimal access and robotic breast surgery depends on continued technological innovation, robust long‐term oncologic outcomes data, standardized training, and certification pathways. Emerging advances in imaging, instrumentation, artificial intelligence, augmented reality, and three‐dimensional modeling are expected to further refine surgical planning, precision, and personalized patient care. International collaboration and consensus guidelines will be essential to ensure the safe and effective integration of endoscopic and robotic techniques into routine breast cancer management for appropriately selected patients [67, 71].

## 7 Conclusion

Collectively, these developments illustrate a fundamental shift in breast cancer surgery—from routine uniform intervention toward an increasingly nuanced, biology‐driven, personalized, and patient‐centered approach to local treatment.

As we look ahead to the next 5 decades, breast cancer management will entail more precision as we proceed further into the molecular era. Surgical staging may be supplemented or even replaced by biological or imaging biomarkers, and artificial intelligence may further refine risk stratification.

## Author Contributions

**Ipshita Prakash:** writing – original draft. **Pooja Ramakant:** writing – original draft. **Sandra Krishnan:** writing – original draft. **Sharon W.W. Chan:** writing – original draft. **Tadahiko Shien:** writing – original draft. **Kavitha Daester:** writing – original draft. **Michael Douek:** writing – original draft. **Mee Hoong See:** writing – original draft. **Jacqueline Jeruss:** writing – review and editing. **Cheng‐Har Yip:** writing – original draft, writing – review and editing. **Ines Buccimazza:** conceptualization, writing – review and editing.

## Funding

The authors have nothing to report.

## Conflicts of Interest

The authors declare no conflicts of interest.

## Data Availability

The data that support the findings of this study are available from the corresponding author upon reasonable request.
